# Quantification and impact of circulating cardiotonic steroids in the RATE-AF randomised trial of patients with atrial fibrillation and heart failure

**DOI:** 10.1186/s12916-025-04476-2

**Published:** 2025-12-29

**Authors:** Ioannis Akoumianakis, Lorna C. Gilligan, Karina V. Bunting, Dannie Fobian, Paulus Kirchhof, Wiebke Arlt, Angela E. Taylor, Davor Pavlovic, Dipak Kotecha, Rate control Therapy Evaluation in permanent Atrial Fibrillation (RATE-AF) trial team

**Affiliations:** 1 Department of Cardiovascular Sciences, University of Birmingham, Birmingham, UK; 2 Department of Cardiology, University Hospitals Birmingham NHS Foundation Trust, Birmingham, UK; 3 Department of Metabolism and Systems Research, University of Birmingham, Birmingham, UK; 4 Department of Cardiology and German Center for Cardiovascular Research (DZHK), University Heart and Vascular Center Hamburg, University Hospital Hamburg-Eppendorf, Hamburg, Germany; 5 MRC Laboratory of Medical Sciences, Faculty of Medicine, Imperial College London, London, UK; 6 NIHR Birmingham Biomedical Research Centre, University Hospitals Birmingham NHS Foundation Trust, Birmingham, UK

**Keywords:** Digoxin, Cardiotonic steroids, CTS, Digoxigenin, Digitoxigenin, Ouabain, Atrial fibrillation

## Abstract

**Background:**

The presence and role of endogenous digoxin-like cardiotonic steroids (CTS) in humans is controversial. This study utilises a novel pipeline to quantify CTS and examines their interaction with digoxin within a randomised trial.

**Methods:**

The RAte control Therapy Evaluation in permanent Atrial Fibrillation (RATE-AF) trial randomised patients with permanent AF and symptoms of heart failure to low-dose digoxin or beta-blocker therapy; clinicaltrials.gov NCT02391337. Circulating CTS were detected and quantified using a new ultra-high-performance liquid chromatography tandem mass spectrometry (LC–MS/MS) pipeline.

**Results:**

All 160 participants of the RATE-AF trial were included, with mean age 76 years (SD 8) and 46% women. Endogenous CTS detected and quantified in baseline samples included digoxigenin and digitoxigenin, plus low or unquantifiable levels of ouabain, telocinobufagin, cinobufagin, marinobufagenin, bufalin, cinobufotalin, dihydroouabain, and ouabagenin. Compared to beta-blockers, patients randomised to digoxin had better functional outcomes at 12 months for heart failure (− 0.57 New York Heart Association class, 95% CI − 0.82 to − 0.32; *p* < 0.001) and atrial fibrillation (odds ratio 2.24 for a two-class improvement in modified European Heart Rhythm Association class, 95% CI 1.43–3.84; *p* < 0.001), with lower NT-pro-B-type natriuretic peptide (geometric mean ratio 0.78, 95% CI 0.61 to 0.99; *p* = 0.006). No interactions were observed for any baseline CTS with each outcome. Digoxin was associated with fewer adverse events (odds ratio 0.16, 95% CI 0.07–0.34; *p* < 0.001), again without any interaction from circulating CTS. Digoxin levels by LC–MS/MS were strongly correlated with measurement by a clinical immunoassay (*r* = 0.87; *p* < 0.001), and treatment with digoxin did not affect CTS concentrations at 6-month follow-up.

**Conclusions:**

A range of CTS are detected in the circulation of patients with atrial fibrillation and heart failure. Within this randomised trial but limited by low circulating levels, CTS do not appear to interact with the ability of digoxin to improve wellbeing compared to conventional first-line treatment with beta-blockers.

**Graphical Abstract:**

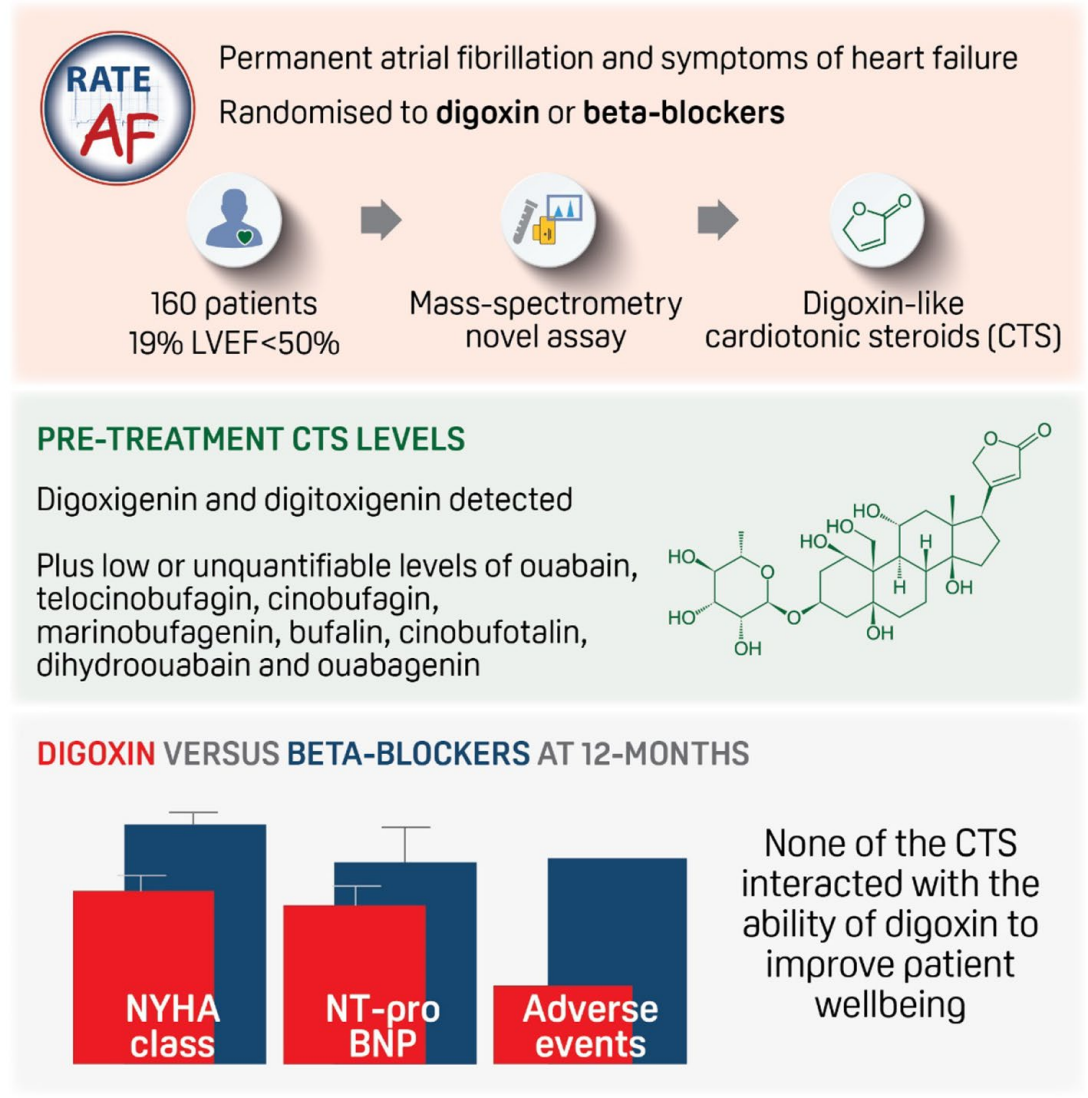

**Supplementary Information:**

The online version contains supplementary material available at 10.1186/s12916-025-04476-2.

## Background

Digoxin has long been used in patients with heart failure (HF) and then atrial fibrillation (AF), given its ability to inhibit the Na + /K + ATPase (NKA), decelerate cardiomyocyte repolarisation, and enhance contractility [1, 2]. Although there is no significant impact on all-cause mortality in randomised controlled trials, in patients with HF digoxin reduces HF-related death, HF-related hospitalisation, and all-cause hospitalisation compared to placebo [3, 4]. Digoxin has a narrow therapeutic range and so has traditionally been used as a second-line therapy in patients with multiple comorbidities, or those with inadequate response or intolerance to beta-blockers. The RAte control Therapy Evaluation in permanent Atrial Fibrillation (RATE-AF) trial showed that low-dose digoxin as an initial strategy for rate control in patients with permanent AF and symptoms of HF was equivalent to beta-blockers for physical-related quality of life, and superior to beta-blockers for a range of other functional and clinical outcomes [5], including health-related cost [6].

Cardiotonic steroids (CTS) comprise a family of digoxin-like molecules which are postulated to originate from the hypothalamus and adrenal glands. CTS, including endogenously produced cardenolides (such as ouabain and digoxin-like compounds) and bufadienolides (such as marinobufagenin and bufalin), have emerged as an important class of circulating hormones with the capacity to modulate physiology [7]. CTS exert their primary effects via reversible inhibition of the NKA, an essential enzyme in virtually all cell types that maintains membrane potential, regulates cell volume, and governs intracellular sodium and calcium handling. Through this inhibition, CTS can directly influence cardiac contractility, vascular tone, neurohormonal activation, and renal tubular sodium reabsorption, and may activate pro-hypertrophic and fibrotic signalling pathways. Elevated circulating levels of several endogenous CTS have been reported in experimental and human studies [7–9], correlating with clinical outcomes [10, 11]. Given the potential for ouabain and other CTS to interfere with the action of digoxin [12], we postulated that varying levels of endogenous CTS could modulate or compete with digoxin’s effects in patients with AF and HF. This study applies high-fidelity mass spectrometric quantification of multiple endogenous CTS in a well-characterised cohort of patients within the setting of a randomised trial, enabling the first systematic evaluation of their concentration, relationship to clinical outcomes, and potential interaction with digoxin therapy.

## Methods

### Study participants

The RATE-AF trial randomised patients in need of rate control to low-dose digoxin or beta-blockers (bisoprolol, or alternative agents if intolerance). Approval was obtained from the East Midlands–Derby research ethics committee (16/EM/0178), the Health Research Authority (IRAS 191437), and the Medicines and Healthcare Products Regulatory Agency. The trial was publicly funded by the UK National Institute for Health & Care Research (NIHR; CDF-2015–08–074) and prospectively registered with clinicaltrials.gov (NCT02391337) and clinicaltrialsregister.eu (2015–005043-13).

Inclusion criteria were (1) age 60 years or older, (2) permanent AF requiring rate control intervention from a clinician’s perspective, and (3) symptoms of breathlessness equivalent to New York Heart Association (NYHA) class II or above. There were limited exclusion criteria to provide a cohort representative of usual clinical practice (Additional file 1: Table S1). Of note, there were no exclusions based on left ventricular ejection fraction (LVEF).

After obtaining written informed consent, participants were randomised in a 1:1 ratio to either digoxin or bisoprolol using a computer-generated minimisation algorithm to ensure balance between the treatment groups for baseline modified European Heart Rhythm Association (mEHRA) class and gender. Allocation was concealed until the baseline assessment was complete; thereafter, it was an open-label trial with blinded endpoints. The trial rationale, design, and protocol have previously been published, which included this secondary analysis of outcomes according to CTS levels in a prospectively designed sub-study of the RATE-AF trial [5, 13].

### Cardiotonic steroid sub-study

The CTS work was funded by the British Heart Foundation (PG/17/55/33087) and utilised blood samples taken at the RATE-AF baseline visit (*n*=160) and the 6-month follow-up visit (*n*=150). All randomised participants agreed with optional consent for storage and processing of plasma. Pre-specified primary outcomes for this sub-study were NYHA class, mEHRA score, N-terminal pro-B-type natriuretic peptide (NT-proBNP), and adverse events at 12 months (*n*=145). Adverse events were collected at each study visit by interview and medical record review to cover all items listed on the summary of product characteristics for each drug. All serious adverse events and incident cardiovascular events underwent a process of independent adjudication, described in more detail previously [6]. Pre-specified secondary outcomes at 6-month follow-up were changes in CTS concentration after treatment, correlation of spectrometry-derived digoxin levels with a clinical immunoassay, and analysis of 6-month outcomes (mEHRA score, NYHA class, NT-proBNP, and adverse events). Study staff working on the CTS study were blinded to the randomised allocation.

### Mass spectrometry

An ultra-high-performance liquid chromatography tandem mass spectrometry (UHPLC-MS/MS) method was used to quantify CTS in plasma after optimisation and validation [13].

In brief, samples were prepared using a 2-mL square well 96-well plate (Porvair Sciences), with 500 μL of each plasma or calibrant/quality control sample mixed with isotopically labelled internal standards. After protein precipitation using zinc sulphate, samples were extracted using solid phase extraction (Thermo SOLA SPE 10 mg/2 mL). The eluate was dried under a nitrogen stream and reconstituted in 200 µL of 10% (v/v) UHPLC-grade methanol (Biosolve) in UHPLC-grade water prior to analysis. Each plate of samples included quality controls to ensure satisfactory extraction and quantification, prepared by spiking a surrogate plasma matrix (phosphate buffered saline with 0.1% bovine serum albumin) at three concentrations (0.3, 1.5, and 15 ng/mL) representing the approximate literature-quoted concentration ranges for ouabain in human circulation. Additionally, serum (Sigma-Aldrich) was used as a biological quality control, spiked at 2 ng/mL with a mix of CTS and bisoprolol.

Chromatography was performed on an ACQUITY UHPLC system (Waters, UK) using a CORTECS T3 1.6 μm 2.1 × 50 mm column at 35 °C. Twenty microlitres of the reconstituted sample was injected. The CTS were chromatographically resolved using a novel convex gradient profile of water (0.1% formic acid) and methanol (0.1% formic acid), with post-column infusion of lithium chloride (LiCl) to form lithium adducts. The UHPLC eluate combined with the post-column LiCl was directed into a Xevo-XS mass spectrometer (Waters, UK) using electrospray ionisation and positive ion mode. CTS were quantified based on their lithium adducts with two mass transitions recorded for each: a quantifier and qualifier. The total run time was 13.5 min.

### Quantification

CTS (ouabagenin, dihydroouabain, ouabain, digoxigenin, marinobufagenin, cinobufotalin, digitoxigenin, telocinobufagin, bufalin, and cinobufagin), digoxin, and bisoprolol were quantified relative to a calibration series ranging from 0.025 to 25 ng/mL. TargetLynx software was used for data processing and quantification. The lower limit of quantification (LLOQ) ranged from 0.025 to 0.1 ng/mL. CTS detected above the LLOQ were quantifiable, those at concentrations below the LLOQ were detectable but not accurately quantifiable, and where no peak was observed the CTS were classed as undetectable. For regression purposes, detectable but not quantifiable CTS were assigned half the value of the lowest detectable concentration for each individual CTS.

### Statistical analysis

Summary results are presented as number and percentage, mean and standard deviation (SD), or median and interquartile range (IQR), as appropriate. To retain the unbiased randomised approach, all analyses used intention-to-treat (with patients in each group categorised by their randomised therapy regardless of treatment use or withdrawal) and with no imputation of missing data. All variables were tested for normality and log-transformed prior to analysis where appropriate. Correlations between variables of interest were evaluated by Pearson’s or Spearman coefficients, and agreement using Bland–Altman plots. Continuous variables are presented as means with standard deviation or medians with interquartile range. Odds ratios (OR) were determined by regression analysis, adjusted for baseline age, sex, LVEF, NYHA class, mEHRA class, estimated glomerular filtration rate (eGFR), and the baseline value of each endpoint of interest. The influence of each baseline CTS on each of the outcomes was evaluated by adding each CTS to the regression models along with an interaction term for treatment allocation. NT-proBNP and NYHA were used as continuous dependent variables in linear regression, whilst mEHRA and adverse events were used as binary variables (mEHRA coded as a 2-class improvement from baseline). Adjusted treatment effects are presented with 95% confidence intervals (CI), with interaction *p* values for each CTS. Regression models were assessed for additional leveraging variables and tested for fitting and potential interactions to assure there were no assumption violations. The effect of digoxin treatment on 6-month CTS levels are presented as standardised beta coefficients determined with linear regression models for each CTS, with baseline CTS values and treatment allocation used as adjusting covariates. Post hoc sensitivity analyses were performed for (1) NYHA and mEHRA using ordinal regression and (2) treatment interactions for digoxigenin, digitoxigenin, and cinobufagin restricted to participants with quantifiable CTS levels. All analyses were performed with STATA version 17 (StataCorp LLC, TX, USA), with two-sided *p* value < 0.05 denoting statistical significance.

## Results

### Patient characteristics

One hundred and sixty patients were randomised and received their allocated medication (80 digoxin and 80 beta-blockers; Additional file 1: Fig. S1). No patients had exposure to digoxin in the month before enrolment. Baseline characteristics are shown in Table 1, with overall mean age 75.6 years (SD 8.2), 46.3% women, the majority with moderate functional impairment due to AF or HF symptoms, and LVEF < 50% in 18.8%. The mean dosage of study drugs after uptitration were 161 µg/day for digoxin (SD 55 µg/day) and 3.2 mg/day for bisoprolol (SD 1.8 mg/day), with 7 patients using alternative beta-blockers.

**Table 1:** Baseline characteristics

	**All (*n* = 160)**	**Digoxin (*n* = 80)**	**Beta-blockers (*n* = 80)**
**Demographics**			
Age, mean years (SD)	75.6 (8.2)	74.5 (8.3)	76.8 (8.1)
Women, *n* (%)	74 (46.3%)	36 (45.0%)	38 (47.5%)
**Comorbidities**			
Hypertension, *n* (%)	116 (72.5%)	56 (70.0%)	60 (75.0%)
Airway disease, *n* (%)	41 (25.6%)	24 (30.0%)	17 (22.5%)
Diabetes, *n* (%)	38 (23.8%)	16 (20.0%)	22 (27.5%)
Stroke/TIA, *n* (%)	28 (17.5%)	12 (15.0%)	16 (20.0%)
Prior hospitalisation for HF/AF, *n* (%)	31 (19.4%)	16 (20.0%)	15 (18.8%)
**HF metrics**			
Previous diagnosis of heart failure, *n* (%)	59 (36.9%)	35 (43.8%)	24 (30.0%)
Signs of HF^a^, *n* (%)	84 (52.5%)	49 (61.3%)	35 (43.8%)
NT-proBNP, median (IQR) pg/mL	1057 (778)	1095 (802)	1041 (727)
LVEF, mean (SD) %	56.9 (9.7)	56.2 (8.8)	57.6 (10.5)
NYHA functional class, *n* (%)			
* I (no limitation)*	0 (0.0%)	0 (0.0%)	0 (0.0%)
* II (slight limitation)*	99 (61.9%)	46 (57.5%)	53 (66.3%)
* III (marked limitation)*	56 (35.0%)	32 (40.0%)	24 (30.0%)
* IV (severe limitation)*	5 (3.1%)	2 (2.5%)	3 (3.8%)
Current ACEi/ARB, *n* (%)	94 (49.3%)	49 (61.3%)	45 (56.3%)
Current use of diuretics, *n* (%)	49 (30.6%)	23 (28.8%)	26 (32.5%)
**AF metrics**			
Prior antiarrhythmics, *n* (%)	13 (8.1%)	5 (6.3%)	8 (10.0%)
Previous cardioversion, *n* (%)	15 (9.4%)	6 (7.5%)	9 (11.3%)
Previous ablation, *n* (%)	3 (1.9%)	2 (2.5%)	1 (1.3%)
mEHRA functional class, *n* (%)			
* 1 (no symptoms)*	0 (0.0%)	0 (0.0%)	0 (0.0%)
* 2a (mild symptoms, not troublesome)*	6 (3.8%)	3 (3.8%)	3 (3.8%)
* 2b (moderate symptoms, troublesome)*	74 (46.3%)	34 (42.5%)	40 (50.0%)
* 3 (severe symptoms, affects daily activity)*	65 (40.5%)	38 (47.5%)	27 (33.8%)
* 4 (disabling symptoms, discontinue activity)*	15 (9.4%)	5 (6.3%)	10 (12.5%)
**Clinical measurements**			
Heart rate, mean (SD) beats/minute	99.7 (18.0)	100.1 (16.8)	99.2 (19.2)
Systolic blood pressure, mean (SD) mmHg	135.6 (16.1)	134.2 (14.7)	137.1 (17.5)
Creatinine, median (IQR) mg/dL	0.97 (0.32)	0.96 (0.30)	0.98 (0.34)
6-min walk distance, median (IQR) m	321 (309)	321 (299)	330 (360)

*AF *atrial fibrillation, *ACEi *angiotensin converting enzyme inhibitor, *ARB *angiotensin receptor blocker, *HF *heart failure, *IQR *interquartile range, *LVEF *left ventricular ejection fraction, *mEHRA *modified European Heart Rhythm Association score, *NT-proBNP *N-terminal pro-brain natriuretic peptide, *NYHA *New York Heart Association, *SD *standard deviation, *TIA *transient ischaemic attack

^a^As determined by the clinical investigator, including lung crepitations, peripheral oedema, raised jugular venous pressure, and abnormal heart sounds

### Detection of circulating cardiotonic steroids

**Fig. 1:**
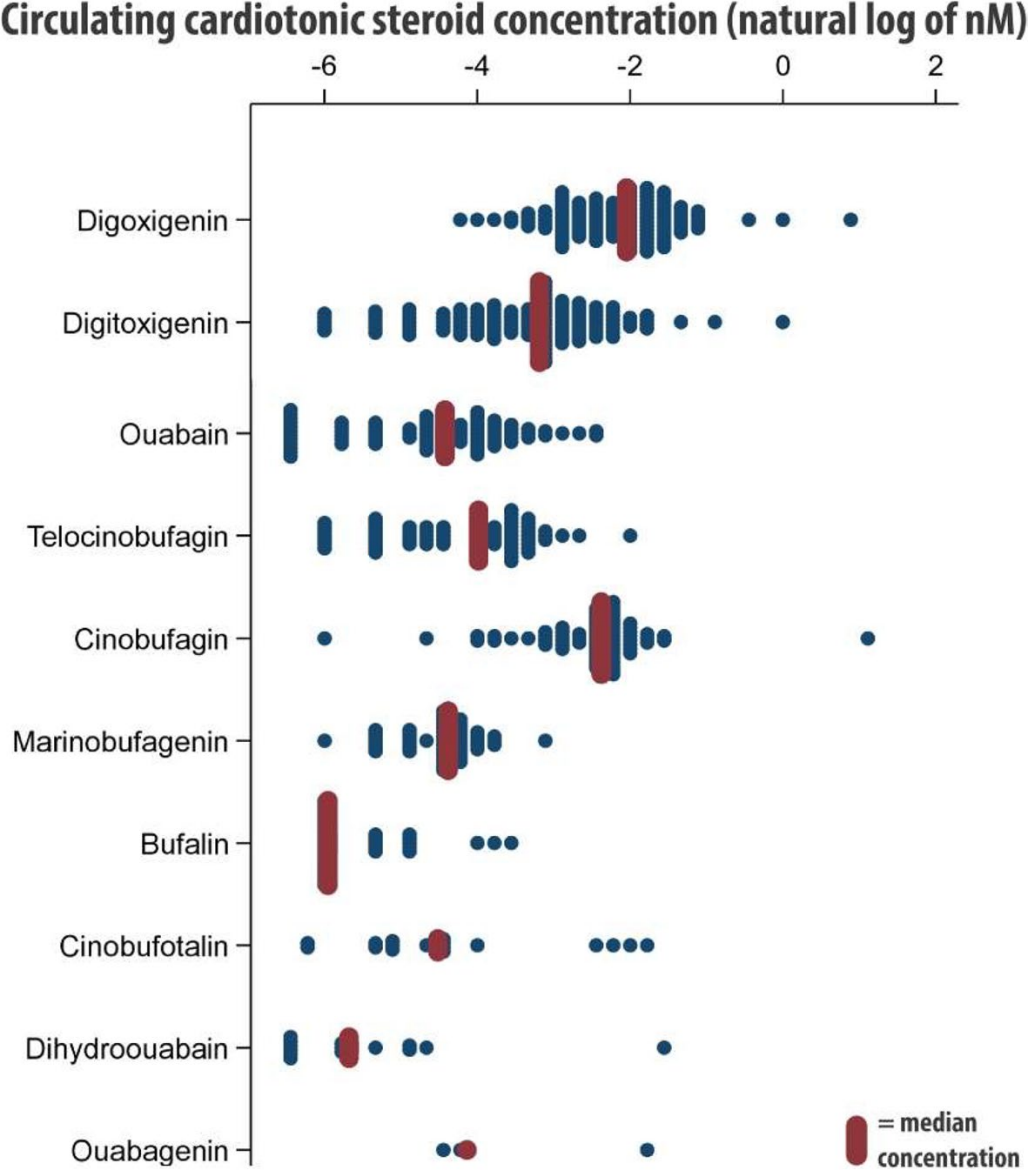
Cardiotonic steroid levels at trial baseline. Distributional dot plot of baseline circulating levels of cardiotonic steroids (CTS) in RATE-AF trial participants randomised to digoxin, presented in logarithmic scale. Median concentrations for each CTS are also highlighted in red. Only cases with detected values are included in the plot

**Fig. 2:**
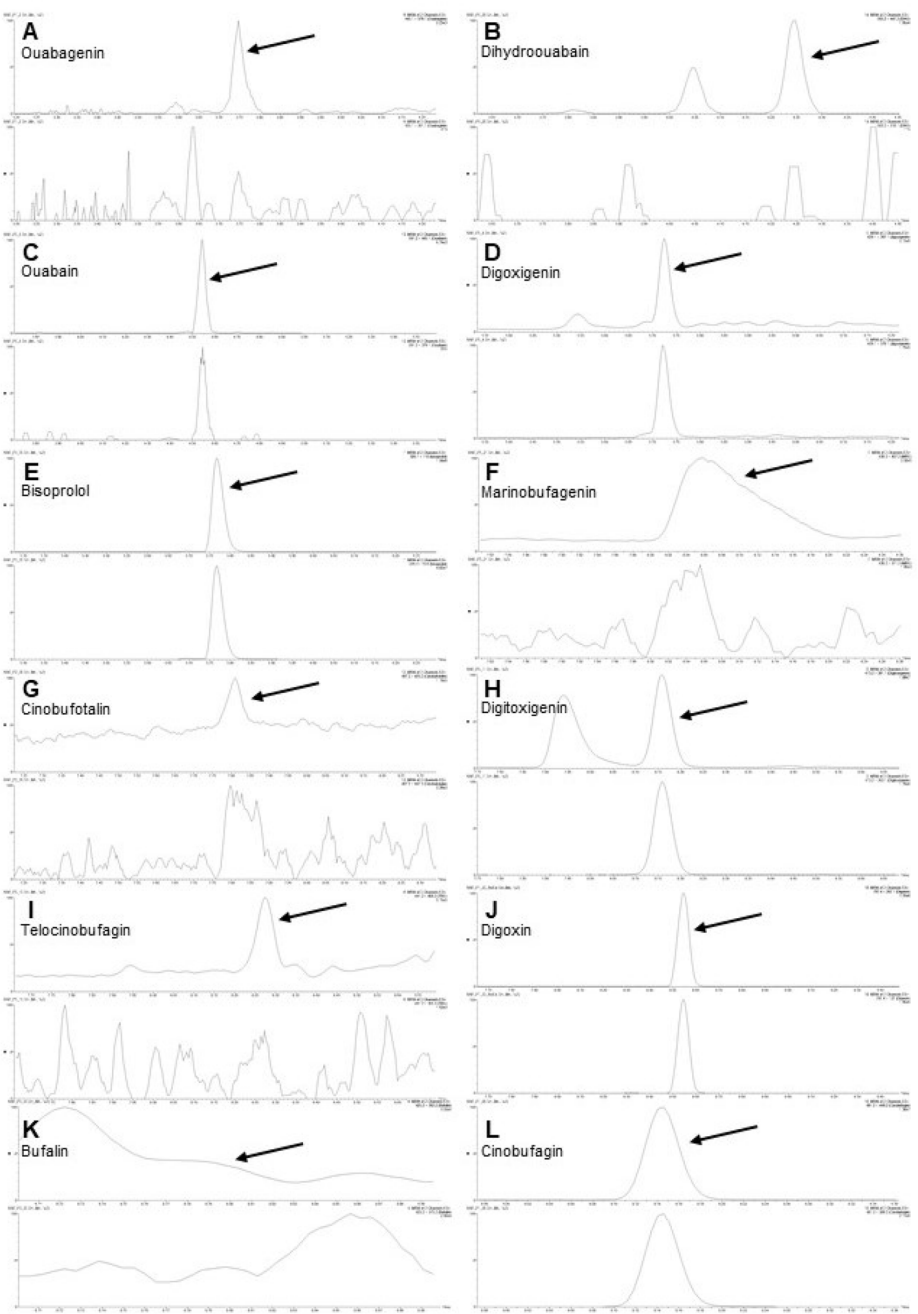
Representative examples of cardiotonic steroid mass spectrometry chromatograms. Chromatograms of quantifier and qualifier peaks of each cardiotonic steroid (CTS) and bisoprolol to demonstrate the mass spectrometry method. Each CTS is from a different patient sample. The arrow points to the CTS quantifier peak for **A** ouabagenin; **B** dihydroouabain (two peaks, with only the second peak used in analysis); **C** ouabain; **D** digoxigenin; **E** bisoprolol; **F** marinobufagenin; **G** cinobufotalin; **H** digitoxigenin (first peak is a matrix effect); **I** telocinobufagin; **J** digoxin; **K** bufalin; and **L** cinobufagin (**G** and **K** are below the LLOQ). Qualifier transitions were not always detectable (**A**, **B**, **G**, **I**, **K**) due to the low concentrations of circulating CTS and the dominance of the Li-adduct quantifier ion

A range of circulating CTS were detected by mass spectrometry: digoxigenin, digitoxigenin, ouabain, telocinobufagin, cinobufagin, marinobufagenin, bufalin, cinobufotalin, dihydroouabain, and ouabagenin. In a large proportion of samples (> 60%), endogenous CTS concentrations were detectable but below the lower limit of quantification (Table 2). Relative distributions for detectable CTS are presented in Fig. 1 and representative mass spectrometry traces in Fig. 2. Digoxigenin (median concentration 0.175 nM) and digitoxigenin (median concentration 0.104 nM) were the most abundant CTS at baseline. Ouabain had the lowest absolute molar concentration, whilst dihydroouabain and ouabagenin tended to be consistently undetectable at baseline (Table 2). Correlations between the different CTS were highly variable (Additional file 1: Fig. S2).

**Table 2:** Circulating cardiotonic steroid measurements

	**Patients with detectable CTS, *n* (%)**	**Patients with quantifiable CTS, *n* (%)**	**Circulating levels in patients with quantifiable CTS, median nM (IQR)**	**Lower limit of quantification, nM**
***Baseline***				
Digitoxigenin	126 (78.8%)	34 (21.3%)	0.104 (0.054)	0.067
Digoxigenin	118 (73.8%)	60 (37.5%)	0.175 (0.082)	0.128
Ouabain	77 (48.10%)	7 (4.4%)	0.068 (0.035)	0.043
Telocinobufagin	68 (42.5%)	1 (0.6%)	n/a	0.124
Cinobufagin	67 (41.9%)	54 (33.8%)	0.103 (0.033)	0.057
Marinobufagenin	49 (30.7%)	1 (0.6%)	n/a	0.063
Bufalin	34 (21.3%)	0 (0.0%)	n/a	0.065
Cinobufotalin	17 (10.6%)	4 (2.5%)	0.156 (0.051)	0.055
Dihydroouabain	14 (8.8%)	1 (0.6%)	n/a	0.085
Ouabagenin	3 (1.9%)	0 (0.0%)	n/a	0.228
***6 months***				
Digitoxigenin	124 (82.7%)	29 (19.4%)	0.083 (0.069)	0.067
Digoxigenin	111 (74.0%)	52 (34.7%)	0.193 (0.074)	0.128
Ouabain	80 (53.3%)	3 (2.0%)	0.046 (0.010)	0.043
Cinobufagin	70 (46.7%)	55 (36.7%)	0.094 (0.026)	0.057
Telocinobufagin	61 (40.7%)	1 (0.7%)	n/a	0.124
Marinobufagenin	35 (23.3%)	1 (0.7%)	n/a	0.063
Bufalin	27 (18.0%)	0 (0.0%)	n/a	0.065
Cinobufotalin	19 (12.7%)	6 (4.0%)	0.125 (0.052)	0.055
Dihydroouabain	16 (10.7%)	1 (0.7%)	n/a	0.085
Ouabagenin	0 (0.0%)	0 (0.0%)	n/a	0.228

*CTS *cardiotonic steroid

### Impact of circulating cardiotonic steroids on the efficacy of digoxin

**Fig. 3:**
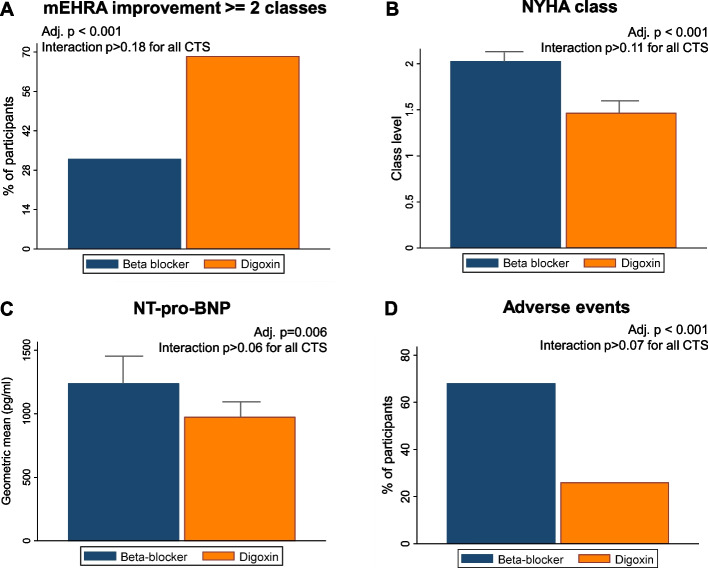
Digoxin vs beta-blockers and cardiotonic steroid interaction. Quantitative effects of digoxin versus beta-blocker treatment and CTS interaction for **A** modified European Heart Rhythm Association (mEHRA) functional class; **B** New York Heart Association (NYHA) functional class; **C** N-terminal pro-B-type natriuretic peptide (NT-proBNP); and **D** adverse events. *p* values are derived from regression models adjusted for age, sex, estimated glomerular filtration rate, baseline left ventricular ejection fraction, baseline NYHA class, baseline mEHRA score, and baseline value of the dependent variable where appropriate

The mean difference in NYHA class at 12 months was − 0.57 favouring digoxin after adjustment for baseline values (95% CI − 0.82 to − 0.32, *p* < 0.001) (Table 3 and Fig. 3). The effect of digoxin was independent of baseline CTS concentrations, with interaction *p* values being non-significant for each CTS tested (Additional file 1: Table S2). A two or more class mEHRA improvement occurred in 50 patients randomised to digoxin (68.4%) versus 23 for beta-blockers (31.9%); OR 2.24, 95% CI 1.43 to 3.84, *p* < 0.001, with no interaction for baseline CTS.

**Table 3:** Effect of digoxin versus beta-blocker treatment

Outcome	Baseline		12 months		Adjusted effect, digoxin vs beta-blocker (95% CI)	Adjusted *p* value
	**Digoxin (*n* = 80)**	**Beta-blockers (*n* = 80)**	**Digoxin (*n* = 73)**	**Beta-blockers (*n* = 72)**		
NYHA class, mean (SD)	2.45 (0.75)	2.38 (0.77)	1.47 (0.65)	2.03 (0.60)	Mean difference − 0.57 (− 0.82 to − 0.32)*	< 0.001
mEHRA two or more class improvement, *n* (%)	n/a	n/a	50 (68.4%)	23 (31.9%)	Odds ratio 2.24 (1.43 to 3.84)†	< 0.001
NT-proBNP, median (IQR)	1095 (812)	1041 (728)	960 (905)	1250 (1043)	Geometric means ratio 0.78 (0.61 to 0.99)	0.006
Adverse events, *n* (%)	n/a	n/a	20 (27.4%)	51 (70.8%)	Odds ratio 0.16 (0.07 to 0.34)	< 0.001

*CI *confidence interval, *IQR *interquartile range, *mEHRA *modified European Heart Rhythm Association, *NT-proBNP *N-terminal pro-B-type natriuretic peptide, *NYHA *New York Heart Association, *SD *standard deviation

*p* values are derived from regression models (linear for NYHA class and NT-proBNP, logistic for mEHRA 2-class improvement and adverse events), adjusted for age, sex, estimated glomerular filtration rate, baseline left ventricular ejection fraction, baseline NYHA class, baseline mEHRA score, and baseline value of dependent variable where appropriate

^*^For NYHA analysed using ordinal logistic regression, the adjusted odds ratio was 0.10 per class favouring digoxin (95% CI 0.04–0.23; *p* < 0.001). †For mEHRA analysed using ordinal logistic regression, the adjusted odds ratio was 0.17 per class favouring digoxin (95% CI 0.08–0.33; *p* < 0.001)

NT-proBNP concentrations reduced from baseline to 12 months in patients randomised to digoxin and increased with beta-blockers. The adjusted geometric means ratio was 0.78 (95% CI 0.61 to 0.99, *p* = 0.006), independent from baseline CTS concentrations.

A total of 20 patients (28.4%) experienced 29 adverse events with digoxin, compared to 51 patients (70.8%) with 142 events for beta-blockers; OR 0.16 (95% CI 0.07 to 0.34, *p* < 0.001). Serious adverse events were experienced by 13 patients (17.8%; 16 events) for digoxin and 21 patients (29.2%; 37 events) for beta-blockers. The lower rate of safety events in those randomised to digoxin was independent of baseline CTS levels (all interaction *p* values non-significant).

Outcomes at the 6-month secondary outcome timepoint followed the same pattern (Additional file 1: Table S3), with no interaction noted from any baseline CTS (Additional file 1: Table S4).

### Change in circulating cardiotonic steroids with treatment

CTS concentrations at 6 months adjusted for baseline values were unchanged after randomised treatment allocation to either digoxin or beta-blockers (Additional file 1: Table S4), with digoxigenin and digitoxigenin remaining the most consistently detectable and quantifiable CTS.

### Digoxin levels using spectrometry versus clinical immunoassay

At 6 months, patients randomised to digoxin had a mean digoxin level of 0.78 ng/mL quantified by the clinical immunoassay (SD 0.31 ng/mL) and 0.89 ng/mL using the novel mass spectrometry assay (SD 0.43 ng/mL). There was a strong correlation and agreement between the immunoassay and mass spectrometry values (Pearson’s *r* = 0.871, *p* < 0.001; Additional file 1: Fig. S3).

## Discussion

This study is the first to provide a comprehensive characterisation of circulating CTS concentrations in patients with AF and HF, using a novel high-performance mass spectrometry assay. Contrary to prior reports that have mostly used immunoassays, we demonstrate that many of the endogenous CTS are at very low or detectable but unquantifiable levels, including ouabain which has been the most studied CTS in animal models. Furthermore, this is the first study to address the interaction of these ‘digoxin-like’ endogenous steroids on the clinical efficacy of digoxin treatment within a randomised trial. None of the CTS interacted with the ability of digoxin to improve patient outcomes in this population.

CTS comprise a family of steroids which have received increasing attention due to their potential biological effects. They are classified into cardenolides and bufadienolides based on their chemical structure [9], although in this study we did not see consistent correlations across CTS using this classification. The assumed roles of CTS include renal salt handing and fluid balance [7, 14], as well as myocardial contractility and heart rate regulation [7, 9, 15]. Consequently, they are implicated in chronic kidney disease [16, 17], hypertension, HF [7, 9], and possibly AF [9], although reliable and reproducible CTS quantification is lacking. Whilst cardenolides such as ouabain have long been proposed to be present in humans [8, 9, 18, 19], immunoassay-based quantification is limited by cross-reactivity and mass spectrometry studies have questioned their validity [20]. On the other hand, several studies have detected ouabain using mass spectrometry in HF patients, healthy volunteers, and patients with end-stage kidney disease [21–23]. The reason for these discrepancies may be due to technical differences between the studies, but also the impact of different patient cohorts. In vitro studies have demonstrated remarkable ouabain-digoxin antagonism [12], warranting this examination in humans.

Atrial fibrillation is an increasingly common condition [24], with substantial impact on patients and society [25]. We demonstrate that digoxin has a significant beneficial effect on NYHA class, mEHRA score, NT-proBNP levels, and clinical events after 12 months of treatment compared to beta-blocker therapy. The interactions of baseline CTS concentrations did not have any effect on digoxin treatment efficacy, although our analysis is limited by concentrations often being below the assay’s lower limit of quantification. Secondary analysis at the 6-month interim visit yielded consistent results. Given that our study participants had no recent exposure to digoxin treatment, it is likely that the values represent endogenous CTS levels, although biosynthetic pathways are still unclear. The biological roles of CTS may be multifaceted, via pleiotropic ion-independent signal transduction secondary to NKA inhibition [26–28]. These effects may vary between CTS, depending on their NKA affinity [28]. Whilst the action of digoxin is thought to be beneficial for cardiac function, marinobufagenin is elevated in humans with HF, whilst marinobufagenin infusion in mice causes myocardial nitrative stress and ventricular dysfunction [10]. Therefore, CTS comprise a diverse family with potentially diverging roles.

This programme of work was designed to identify patient-level predictors of digoxin response, and whilst CTS were not found to be of value in this regard, it is reassuring that the efficacy of low-dose digoxin is independent from the range of underlying CTS pathways in humans. The lack of change in CTS concentration is supportive of the consistency of the novel spectrometry assay, but we cannot exclude higher digoxin doses having a greater impact on CTS concentration, or conversely higher CTS concentrations having an impact on digoxin treatment. An important complementary finding of our study was that quantification of digoxin using mass spectrometry correlated strongly with measurements from a clinical immunoassay. Whether this correlation would be maintained in the context of higher CTS concentrations has yet to be investigated.

Study strengths included a randomised trial design, results analysed in a blinded fashion, an intention-to-treat approach to avoid bias, and no patients in the beta-blocker group given digoxin during 12-month follow-up. A limitation of this study is the absence of a true control group, as all participants were randomised to either digoxin or beta-blockers. However, this better reflects clinical practice where beta-blockade is usually standard practice, and does not hinder our ability to measure and understand the effects of CTS in secondary analysis of the study outcomes. The RATE-AF trial primary outcome was patient-reported physical quality of life; however, work by the patient and public involvement team identified this as a poor indicator of patient progress [29], hence the pre-specified focus in this sub-study on functional outcomes. There are other important limitations to this work that should be considered. First, the low concentrations of CTS limit the ability to identify weak or small interactions with treatment effect. Our findings should be interpreted in the context of the assay limitations, with over 60% of samples having CTS concentrations below the quantifiable detection threshold. It is possible that the study is not adequately powered to detect interactions between CTS and these outcomes; however, any clinically relevant difference should have been noted with this sample size and methodology. RATE-AF is the only randomised trial available in this population, so further external validation is not feasible. Second, the trial recruited symptomatic patients with AF and HF, with the majority having preserved ejection fraction. Although 19% of patients had LVEF < 50%, the findings may not apply to populations with severely depressed systolic function. Third, the majority of participants were of European descent and hence further study is warranted in other ethnicity groups. Finally, to further elucidate the metabolism and role of CTS, validated mass spectrometry methods, such as that utilised in this study, need to be applied to other patient cohorts, including those with chronic kidney disease and other comorbidities that have an impact on CTS levels.

## Conclusions

This study provides the first comprehensive characterisation of circulating CTS concentrations in humans with AF and HF using a novel mass spectrometry method. Based within a randomised controlled trial, the efficacy of digoxin was unaffected by baseline CTS levels, but these were generally at low or unquantifiable concentrations limiting further conclusions as to their biological importance. Personalising rate control strategies with individual assessment of CTS is unlikely to have clinical value when using low doses of digoxin.

## Supplementary Information

Additional file 1. Tables S1–S5. Table S1 Inclusion and exclusion criteria of the RATE-AF trial. Table S2 Multivariate interaction terms for baseline circulating CTS. Table S3 Effect of digoxin vs beta-blocker treatment on outcomes at 6 months (secondary outcome). Table S4 Interaction of baseline circulating CTS on outcomes at 6 months (secondary outcome). Table S5 Effect of rate control treatment on circulating CTS from baseline to 6 months. Figures S1–S3. Fig. S1 CONSORT flow chart. Fig. S2 Correlation matrix of baseline circulating cardiotonic steroid levels. Fig. S3 Novel mass spectrometry assay versus clinical immunoassay for digoxin.

## Data Availability

The datasets used and/or analysed during the current study are available from the corresponding author on reasonable request after review by the RATE-AF Steering Committee, with any datasets provided restricted to ensure confidentiality of all included participants.

## Abbreviations

AF: Atrial fibrillation
CTS: Cardiotonic steroids
CI: Confidence interval
eGFR: Estimated glomerular filtration rate
HF: Heart failure
IQR: Interquartile range
LVEF: Left ventricular ejection fraction
mEHRA: Modified European Heart Rhythm Association class
NKA: Na + /K + ATPase
NYHA: New York Heart Association class
NT-proBNP: N-terminal pro-B-type natriuretic peptide
RATE-AF: RAte control Therapy Evaluation in permanent Atrial Fibrillation
SD: Standard deviation
UHPLC-MS/MS: Ultra-high-performance liquid chromatography tandem mass spectrometry
