# Supplementary material for: Quantification and impact of circulating cardiotonic steroids in the RATE-AF randomised trial of patients with atrial fibrillation and heart failure
Source: BMC Med. 2025 Dec 29;23:694. doi: 10.1186/s12916-025-04476-2 (PMC12751528; doi:10.1186/s12916-025-04476-2)
Supplement: Supplementary file 1 — Additional file 1. Tables S1–S5. Table S1 Inclusion and exclusion criteria of the RATE-AF trial. Table S2 Multivariate interaction terms for baseline circulating CTS. Table S3 Effect of digoxin vs beta-blocker treatment on outcomes at 6 months (secondary outcome). Table S4 Interaction of baseline circulating CTS on outcomes at 6 months (secondary outcome). Table S5 Effect of rate control treatment on circulating CTS from baseline to 6 months. Figures S1–S3. Fig. S1 CONSORT flow chart. Fig. S2 Correlation matrix of baseline circulating cardiotonic steroid levels. Fig. S3 Novel mass spectrometry assay versus clinical immunoassay for digoxin. [file 12916_2025_4476_MOESM1_ESM.docx]

**Quantification and impact of circulating cardiotonic steroids in the RATE-AF randomised trial of patients with atrial fibrillation and heart failure**

**Additional File 1**

[Table S1: Inclusion and exclusion criteria of the RATE-AF trial 2](#_Toc208411161)

[Table S2: Multivariate interaction terms for baseline circulating CTS 3](#_Toc208411162)

[Table S3: Effect of digoxin vs beta-blocker treatment on outcomes at 6 months (secondary outcome) 4](#_Toc208411163)

[Table S4: Interaction of baseline circulating CTS on outcomes at 6 months (secondary outcome) 5](#_Toc208411164)

[Table S5: Effect of rate control treatment on circulating CTS from baseline to 6 months 6](#_Toc208411165)

[Figure S1: CONSORT flow chart 7](#_Toc208411166)

[Figure S2: Correlation matrix of baseline circulating cardiotonic steroid levels 8](#_Toc208411167)

[Figure S3: Novel mass spectrometry assay versus clinical immunoassay for digoxin 9](#_Toc208411168)

## Table S1: Inclusion and exclusion criteria of the RATE-AF trial

| **Inclusion criteria** |
| --- |
| Adult participants, 60 years old or older |
| Permanent atrial fibrillation, characterised (at time of randomisation) as a physician decision for rate-control with no plans for cardioversion, anti-arrhythmic medication, or ablation therapy |
| Symptoms of breathlessness (New York Heart Association Class II or more) |
| Able to provide written informed consent |
| **Exclusion criteria** |
| Established clinical indication for beta-blocker therapy, e.g. myocardial infarction in the last 6 months |
| Known contraindications for therapy with beta-blockers or digoxin, e.g. a history of severe bronchospasm that would preclude use of beta-blockers, or known intolerance to these medications |
| Baseline heart rate <60 beats/minute |
| History of second or third-degree heart block |
| Supraventricular arrhythmias associated with accessory conducting pathways (e.g. Wolff-Parkinson-White syndrome) or a history of ventricular tachycardia or fibrillation |
| Planned pacemaker implantation (including cardiac resynchronisation therapy), pacemaker-dependent rhythm or history of atrioventricular node ablation |
| Decompensated heart failure (evidenced by need for intravenous inotropes, vasodilators or diuretics) within 14 days prior to randomisation |
| A current diagnosis of obstructive hypertrophic cardiomyopathy, myocarditis or constrictive pericarditis |
| Received or on waiting list for heart transplantation |
| Receiving renal replacement therapy |
| Major surgery, including thoracic or cardiac surgery, within 3 months of randomisation |
| Severe, concomitant non-cardiovascular disease (including malignancy) that is expected to reduce life expectancy |

**Table S2: Multivariate interaction terms for baseline circulating CTS**

| **Cardiotonic steroid** | **NYHA class**  ***Interaction p-value*** | **mEHRA class**  ***Interaction p-value*** | **NTpro-BNP**  ***Interaction p-value*** | **Adverse events**  ***Interaction p-value*** |
| --- | --- | --- | --- | --- |
| Digoxigenin* | 0.69 (0.21) | 0.63 (0.83) | 0.06 (0.80) | 0.23 (0.56) |
| Digitoxigenin* | 0.91 (0.58) | 0.21 (0.18) | 0.56 (0.80) | 0.84 (0.18) |
| Ouabain | 0.64 | 0.44 | 0.76 | 0.23 |
| Telocinobufagin | 1.00 | 0.89 | 0.07 | 0.92 |
| Cinobufagin* | 0.24 (0.52) | 0.48 (0.28) | 0.50 (0.93) | 0.07 (0.30) |
| Marinobufagenin | 0.91 | 0.53 | 0.37 | 0.42 |
| Bufalin | 0.67 | 0.77 | 0.36 | 0.41 |
| Cinobufotalin | 0.83 | 0.67 | 0.91 | 0.60 |
| Dihydroouabain | 0.29 | 0.31 | 0.33 | 0.86 |
| Ouabagenin | 0.12 | 0.99 | 0.55 | 0.99 |

Outcomes are for the primary timepoint of 12-months post-randomisation. * p-values in brackets are for the post-hoc sensitivity analyses restricting to participants with quantifiable CTS.

IQR: Interquartile range; mEHRA: Modified European Heart Rhythm Association; NT-proBNP: N-terminal pro-brain natriuretic peptide; NYHA: New York Heart Association; SD: Standard deviation. Interaction p values are derived from interaction terms for each cardiotonic steroid added to reference regression models for each outcome.

## Table S3: Effect of digoxin vs beta-blocker treatment on outcomes at 6 months (secondary outcome)

| **Outcome** | **Baseline** | | **6 months** | | **Adjusted effect: digoxin vs beta-blocker (95% CI)** | **Adjusted p value** |
| --- | --- | --- | --- | --- | --- | --- |
|  | **Digoxin, baseline (n=80)** | **Beta-blocker, baseline (n=80)** | **Digoxin, 6 months (n=73)** | **Beta-blocker, 6 months (n=72)** |  |  |
| NYHA class, mean (SD) | 2.45 (0.8) | 2.38 (0.8) | 1.53 (0.9) | 2.07 (1.0) | Mean difference -0.57  (-0.81 to -0.33) | <0.001 |
| mEHRA two or more class improvement, n (%) | n/a | n/a | 40 (52.6) | 7 (9.5) | Odds ratio 5.96  (2.79 to 23.92) | <0.001 |
| NTpro-BNP, median pg/mL (IQR) | 1095 (812) | 1041 (728) | 1058 (906) | 1209 (694) | Geometric means ratio 0.83  (0.61 to 1.13) | 0.10 |
| Adverse events, n (%) | n/a | n/a | 13 (17.1) | 44 (59.5) | Odds ratio 0.28  (0.12 to 0.52) | <0.001 |

Adjusted mean changes are calculated as odds ratios for mEHRA two-class improvement & adverse event composite, mean differences for NYHA and geometric means ratio for NTpro-BNP; adjustments are made for age, sex, estimated glomerular filtration rate, baseline NYHA, baseline mEHRA, baseline left ventricular ejection fraction and baseline value of dependent variable (in the case of NT-pro-BNP). P values are calculated from adjusted regression models.

CI: Confidence interval; mEHRA: modified European Heart Rhythm Association; NYHA: New York Heart Association; SD: Standard deviation; IQR: Interquartile range; NT-proBNP: N-terminal pro-brain natriuretic peptide.

## Table S4: Interaction of baseline circulating CTS on outcomes at 6 months (secondary outcome)

| **CTS** | **NYHA class**  ***Interaction p-value*** | **mEHRA class**  ***Interaction p-value*** | **NTpro-BNP**  ***Interaction p-value*** | **Adverse events**  ***Interaction p-value*** |
| --- | --- | --- | --- | --- |
| Digoxigenin | 0.74 | 0.65 | 0.46 | 0.09 |
| Digitoxigenin | 0.61 | 0.27 | 0.97 | 0.81 |
| Ouabain | 0.54 | 0.22 | 0.70 | 0.81 |
| Telocinobufagin | 0.58 | 0.27 | 0.42 | 0.92 |
| Cinobufagin | 0.68 | 0.61 | 0.40 | 0.07 |
| Marinobufagenin | 0.20 | 0.23 | 0.24 | 0.17 |
| Bufalin | 0.81 | 0.68 | 0.88 | 0.64 |
| Cinobufotalin | 0.36 | 0.79 | 0.84 | 0.86 |
| Dihydroouabain | 0.59 | 0.39 | 0.86 | 0.66 |
| Ouabagenin | 0.82 | 0.99 | 0.89 | 0.99 |

Interaction p values are derived from interaction terms for each CTS added to reference regression models for each outcome.

mEHRA: modified European Heart Rhythm Association; NYHA: New York Heart Association; SD: Standard deviation; IQR: Interquartile range; NT-proBNP: N-terminal pro-brain natriuretic peptide.

## Table S5: Effect of rate control treatment on circulating CTS from baseline to 6 months

| **Cardiotonic steroid** | **Rate control treatment effect; Adjusted geometric mean ratio (95% CI) from baseline to 6-months** | **Interaction p-value for rate control on CTS change** |
| --- | --- | --- |
| Digoxigenin | 1.34 (0.77 to 2.34) | 0.14 |
| Digitoxigenin | 1.14 (0.59 to 2.21) | 0.18 |
| Ouabain | 1.01 (0.55 to 1.85) | 0.38 |
| Telocinobufagin | 0.94 (0.58 to 1.52) | 0.80 |
| Cinobufagin | 0.78 (0.39 to 1.56) | 0.94 |
| Marinobufagenin | 1.04 (0.79 to 1.38) | 0.16 |
| Bufalin | 1.04 (0.88 to 1.23) | 0.94 |
| Cinobufotalin | 1.03 (0.67 to 1.57) | 0.29 |
| Dihydroouabain | 1.10 (0.83 to 1.47) | 0.56 |
| Ouabagenin | 1.00 (0.80 to 1.20) | 1.00 |

Treatment effects are presented as geometric mean ratios in all trial participants. Adjusting covariates included treatment allocation (digoxin vs beta-blocker) and baseline cardiotonic steroid values. P values are derived from adjusted linear regression models for 6 months vs baseline for each cardiotonic steroid.

CI: Confidence interval.

## Figure S1: CONSORT flow chart

All participants in the RATE-AF trial provided optional consent to contribute to the cardiotonic steroid sub-study.

## Figure S2: Correlation matrix of baseline circulating cardiotonic steroid levels


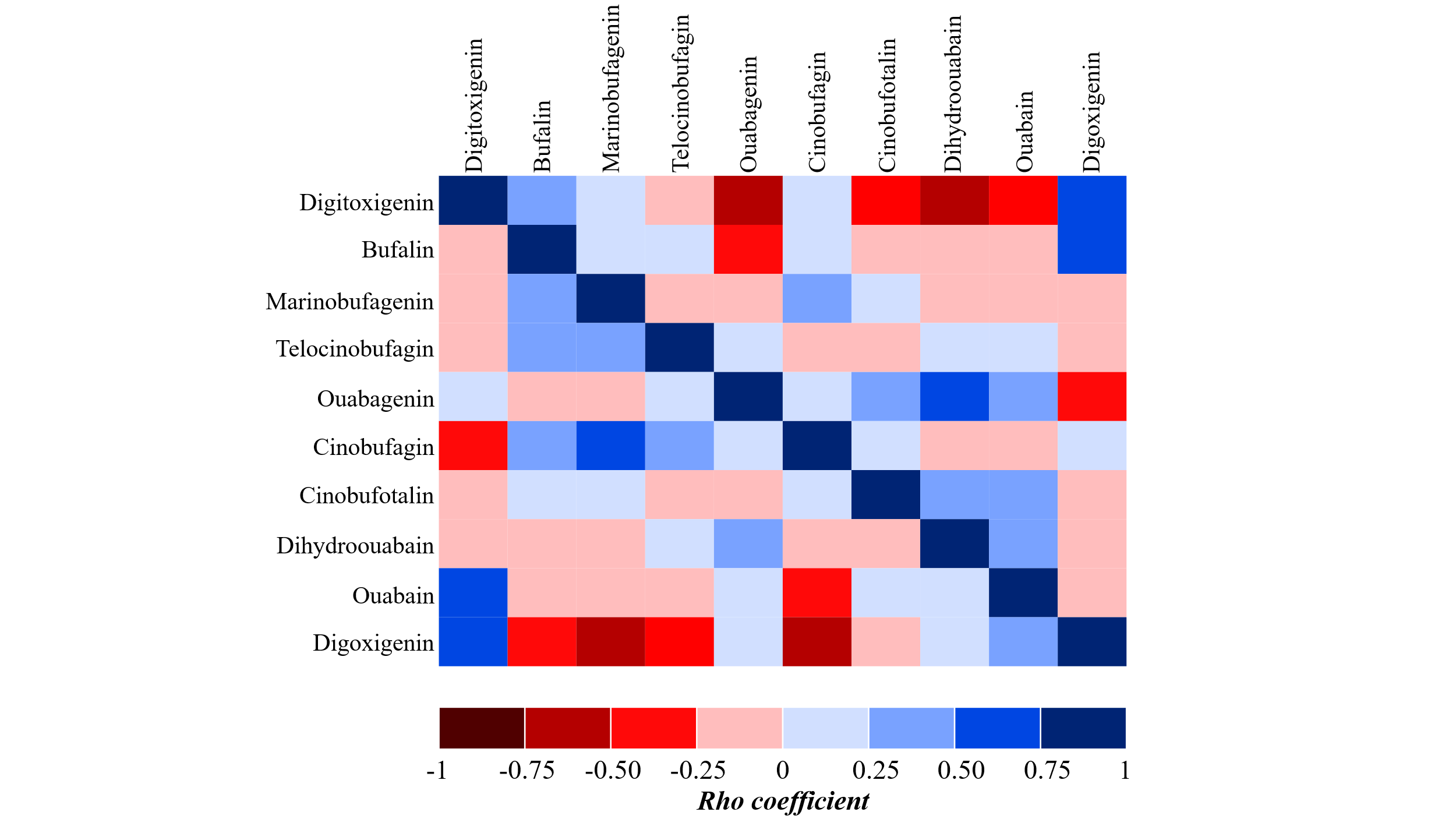


Colour-coded correlation matrix of baseline CTS, assessed by Spearman coefficients.

Digoxigenin, digitoxigenin, dihydroouabain, ouabain and ouabagenin are cardenolide CTS. Bufalin, cinobufagin, cinobufotalin, marinobufagenin and telocinobufagin are bufadienolide CTS.

## Figure S3: Novel mass spectrometry assay versus clinical immunoassay for digoxin


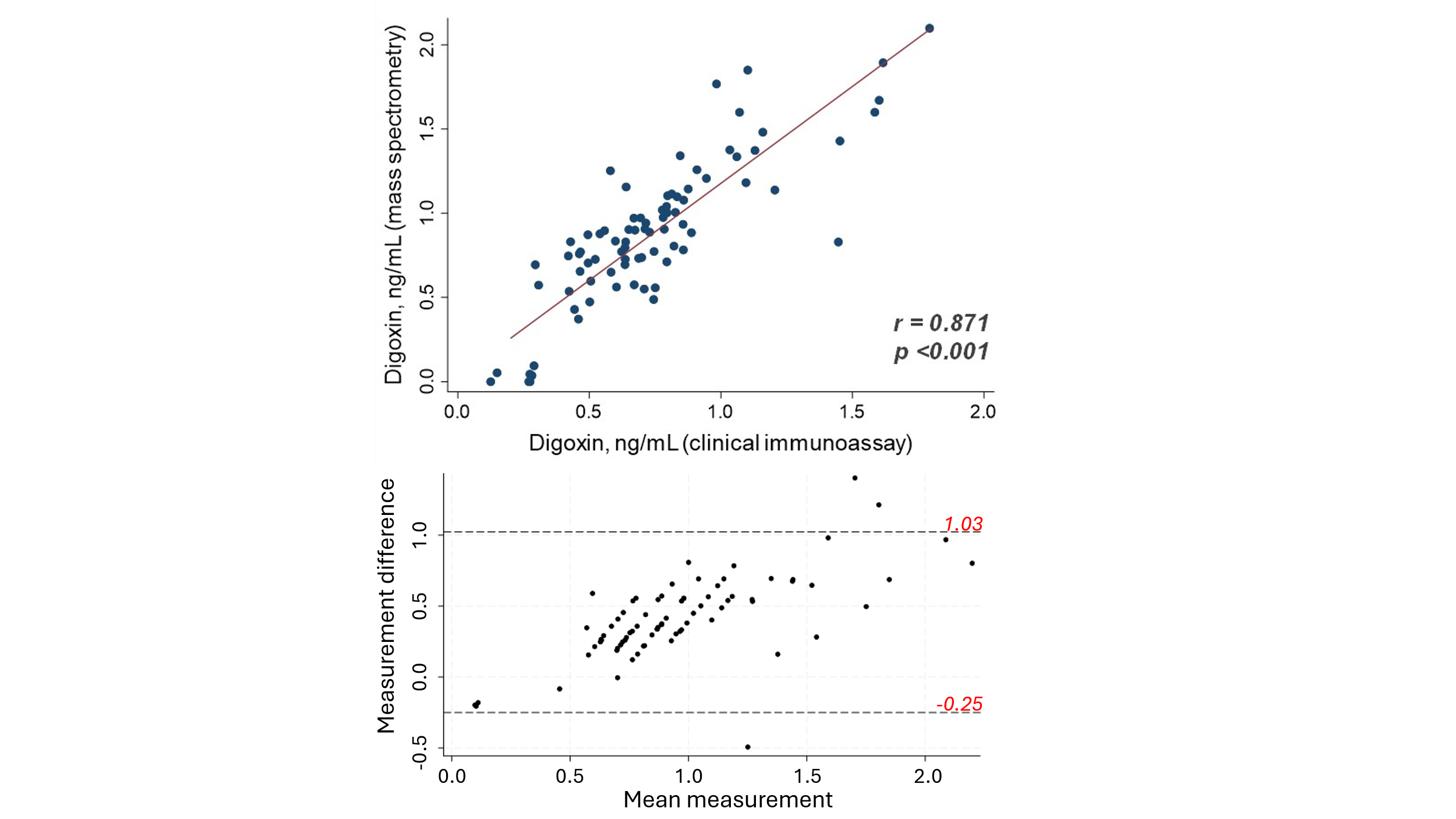


Analysis based on n=73 paired samples. Top panel demonstrates very strong correlation between the mass spectrometry and clinical immunoassay for digoxin serum concentration. Bottom panel demonstrates good agreement between methods by Bland-Altman analysis, with the limits of agreement -0.25 to 1.03 ng/mL.
